# A cross-over trial on soy intake and serum leptin levels in women with metabolic syndrome

**Published:** 2010

**Authors:** Leila Azadbakht, Ahmad Esmaillzadeh

**Affiliations:** a Food Security Research Center, Isfahan University of Medical Sciences, Isfahan, Iran; b Department of Nutrition, School of Public Health, Isfahan University of Medical Sciences, Isfahan, Iran

**Keywords:** Soy Foods, Leptin, Metabolic Syndrome X, Postmenopause

## Abstract

**BACKGROUND:**

Soy consumption may affect serum leptin levels and exert its beneficial effects in this way. The aim of this study was to evaluate the effect of soy consumption on serum leptin levels in postmenopausal women with metabolic syndrome.

**METHODS:**

In this clinical trial, 42 postmenopausal women with metabolic syndrome were included. The patients followed three kinds of diets: control diet (Dietary Approaches to Stop Hypertension= DASH), soy protein diet, or soy nut diet for eight weeks. Serum leptin level was measured by ELISA method.

**RESULTS:**

No significant weight change were seen in patients during three phases of trial. There was no significant difference between the end values of serum leptin concentrations following these diets (Geometric mean ± SD: 16.9 ± 2.5 ng/ml at the end of control diet, 16.1 ± 1.6 ng/ml at the end of soy protein diet, and 15.9 ± 1.7 ng/ml at the end of soy nut diet). Percent difference compared to control for serum leptin levels showed that neither soy protein nor soy nut diets could significantly alter this variable (p = 0.32).

**CONCLUSIONS:**

The results of the present study showed that neither soy protein, nor soy nut could affect weight and serum leptin levels in postmenopausal women with metabolic syndrome.

Metabolic syndrome is a complex of abnormalities in blood pressure, lipids and sugars, and is associated with central adiposity.^1^ Insulin resistance is a key factor in this syndrome.^1^ The prevalence of this syndrome is rising in both developed and developing countries across the world.^2^,^3^ Many environmental and dietary factors might be related to the etiology of this syndrome.^4^

Among dietary factors soy products are reported to have a beneficial role in the management of the metabolic syndrome.^5^ Previous studies have reported a reduction in plasma inflammatory markers,^6^ malondealdehyde^7^ and also improvement in some features of this condition by the use of soy products.^8^ There are different probable action mechanisms for beneficial effects of soy components.^5^ One hypothesis might be related to the serum leptin levels. The anti-atherogenicity or anti-inflammatory effect of soy might be related to its effect on reducing the serum leptin levels.^9^ A systematic review also indicated the associations between leptin and various pathologic components of metabolic syndrome with emphasis on the hypertension, impaired glucose metabolism and pro-atherogenic state in metabolic syndrome.^10^ There is a well-documented effect of esterogen on increasing the leptin production; however, even high levels of isoflavone consumption do not alter leptin concentrations in women.^11^

Findings of the studies on the effect of soy product consumption on serum leptin levels in animal models and humans are inconsistent.^12^–^15^ However, to our knowledge, no study has assessed the effects of soy intake on serum leptin levels among patients with the metabolic syndrome, while it is assumed that serum leptin levels might play a role in different aspects of the disease. Therefore, the present study was conducted to determine the effect of soy protein (in the form of textured soy protein) and soy nut intake on serum leptin levels in postmenopausal women with the metabolic syndrome.

## Methods

### 

#### Participants

In this study, 42 Tehrani women with metabolic syndrome were included and they continued their participation until the end of the research. The sampling method and inclusion and exclusion criteria has been reported elsewhere.^6^–^8^ Informed written consent was signed by all the women participated in this study. Research council and also the ethical committee of the National Nutrition and Food Technology Research Institute of Shaheed Beheshti University of Medical Sciences approved the study.

#### Study Procedures

This study was a randomized cross-over clinical trial. A run-in period was considered for three weeks. After that women were assigned randomly to consume a control diet, DASH diet with soy nut, or DASH diet with soy protein; each diet was continued for eight weeks. So, two wash-out periods of 4 weeks each were considered. During the study, patients had to record their physical activity three days per month and they were asked to maintain a stable level of physical activity.

## Diets

### Three diets were used:

1. **Control Diet:** a DASH diet was considered with 55% of carbohydrates, 17% of protein and 28% of total fat; Na intake was 2400 mg/day.^16^
2. **Diet With Soy Nut:** a DASH diet was also prescribed, but 30 grams of roasted soy nut was considered instead of one exchange of red meat.
3. **Diet With Soy Protein:** a DASH diet was also considered, but 30 grams of soy protein was prescribed instead of one exchange of red meat. No specific form of red meat was prescribed and patients were allowed to use red meat in different forms such as boiled, grilled, etc. The soy protein was prescribed to be soaked for 20-30 minutes and then by adding lemon juice, turmeric and paste for better flavor, they could add it to any kind of food. Soy nut, lemon flavored and without salt, was used as snack. The patients were allowed to use these items in any meal or snack that they wanted during a day. The components of soy protein and soy nut are shown in table 1. These values were obtained by analyzing samples from each product by the standard methods.

**Table 1:** Nutrient composition of soy protein and soy nut used in the intervention

Nutrients/30 g	Soy protein	Soy nut
Protein (g)	15	11.3
Fat (g)	0.3	7
Fiber (g)	10	9
Sodium (mg)	9	10
Total phytoestrogens (mg)	84	102

The calorie was prescribed according to each patient’s individual needs.^17^ Three-day diet records were used for assessing the dietary intake during each month of the study. Analyzing 3-day diet records and also the plasma levels of phytoestrogens showed the diet compliance of the participants.

#### Measurements

Body weight and height were measured by standard methods. Waist circumference (WC) was measured at the narrowest level by an unstretchable tape measure.

All the samples were collected after 24 hours fasting. Serum leptin levels were measured using ELISA (Diaclone Besancon, France). The sensitivity of the assay for leptin was 0.10 ng/ml. Inter-and intra-assay CVs were both less than 10%. Radioimmunoassay was used for measuring serum follicle-stimulating hormone. Franke et al method was used for measuring the plasma phytoestrogen levels.^18^,^19^ Enzymatic reagents were used for assessing the amount of lipid profiles.^20^ The possible change in appetite was also asked by a simple question in all three phases of the study.

#### Statistical Analysis

General linear model (repeated measures analysis of variance) was used to compare means of the variables at the end of each trial. We used the formula [(E-B)/B × 100], for calculating the percent change of serum leptin level. In this formula E was the end of treatment values and B was the baseline values. Percent difference compared to control for serum leptin levels was also determined by the formula [(X-C)/C × 100]. In this formula, C was the end values of control group and X was the end values of soy protein or soy nut. The percent change of serum leptin levels in the three groups was compared using further models adjusted for lipid profile change. Period effect and carryover effects were tested using the appropriate general linear models.

Serum leptin level was a skewed variable. So, we used log-transformed values in all analyses and reported geometric means. Pearson correlation coefficients were used to evaluate the relationship between soy-derived phytoestrogens intake (calculated from self reported soy intake in 3-day diet records) and plasma phytoestrogen levels. All results were considered significant if the two-tailed p value was < 0.05. Statistical analysis was performed using SPSS for Windows version 13.0 (SPSS, Chicago IL) and SAS version 8.2 (SAS Institute Inc, 1999).

## Results

Dietary intake of subjects in each trial period is shown in table 2. There was a significant difference in fat intake of these three periods, which was related to the difference in the fat content of soy products. No significant change in patients’ weight was seen during three phases of trial (Mean ± SD: 70.1 ± 0.9 kg at the end of control diet, 70.7 ± 0.9 kg at the end of soy protein diet, 70.4 ± 0.8 kg at the end of soy nut diet; p = 0.57). Waist circumference values also did not change significantly after three diet periods (Mean ± SD: 91.9 ± 0.8 kg at the end of control diet, 91.5 ± 0.9 kg at the end of soy protein diet, 91.0 ± 1.0 kg at the end of soy nut diet; p = 0.19).

**Table 2:** Dietary intake of participants separately by intervention period

Dietary intakes (/d)	Control* (n = 42)	Soy protein** (n = 42)	Soy nut† (n = 42)	P value††	Wash-out§ (n = 42)
Nutrients					
Energy (Kcal)	2055	2039	2049	0.62	2078
Protein (% of energy)	17	17	17	0.71	15
Total fat (% of energy)	28	25	29	< 0.05	31
Saturated fat (% of energy)	7	5	5	0.61	14
Polyunsaturated fat (% of energy)	8	8	11	< 0.05	7
Monounsaturated fat (% of energy)	10	10	10	0.73	9
Carbohydrate (% of energy)	55	58	57	0.79	54

* Control diet: this diet had one serving of red meat and was rich in fruits, vegetables, whole grains, low-fat dairy products, and low in saturated fat, total fat, cholesterol, refined grains, and sweets. The amount of Na intake was 2400 mg per day (Dietary Approach to Stop Hypertension pattern).

** Soy protein diet: this diet was the same as control diet (DASH diet) but red meat was replaced by soy protein.

† Soy nut diet: this diet was the same as control diet (DASH diet) but red meat was replaced by soy nut.

†† P values for differences among three trial periods (repeated measures analysis of variance)

§ Wash-out: in this period, patients used the same diet they were using before the study.

**Figure 1:**
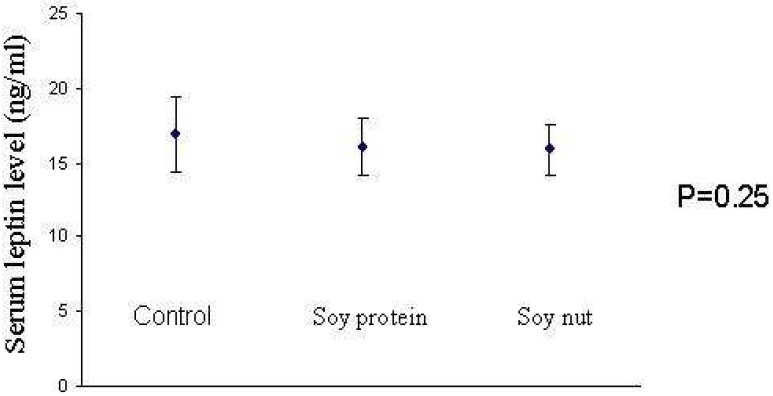
Mean and standard deviation of serum leptin levels at the end of trial in three diet periods Control diet: a DASH diet; Diet with soy nut: a DASH diet also was prescribed but 30 grams of roasted soy nut was considered instead of one exchange of red meat; Diet with soy protein: a DASH diet was also considered but 30 grams of soy protein was prescribed instead. P values were resulted from a repeated measures analysis of variance.

Figure 1 shows the geometric means of serum leptin levels at the end of trial across three diet periods. There was no significant difference between the end values of serum leptin concentrations following these diets.

**Figure 2:**
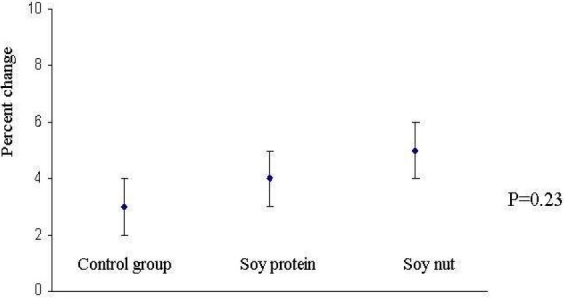
Mean and 95% confidence interval of percent changes in serum leptin levels in three diets: control, soy protein, soy nut. Control diet: a DASH diet; Diet with soy nut: a DASH diet also was prescribed but 30 grams of roasted soy nut was considered instead of one exchange of red meat; Diet with soy protein: a DASH diet was also considered but 30 grams of soy protein was prescribed instead. P values were resulted from a repeated measures analysis of variance.

Figure 2 presents mean and 95% confidence intervals of percent changes of leptin levels across three diet periods. The results were not changed with the adjustment of the means for the alterations in lipid profiles in further models (data not shown).

Neither soy protein nor soy nut consumption significantly changed weight, waist circumference and serum leptin levels compared to the control diet. Percent differences compared to control for serum leptin levels showed that neither soy protein nor soy nut could significantly change this variable (p = 0.32). None of the patients mentioned any change in their appetite or the amount of food intake. Comparing the serum phytoesterogen levels in each group, serum phytoesterogen level in soy groups was higher than the control one (p = 0.04). There was also a significant and direct association between soy intake and the phytoesterogen level (p = 0.04).

## Discussion

The results of the present study on a group of postmenopausal women with the metabolic syndrome indicated that neither soy protein nor soy nut could change the serum leptin levels. The serum leptin levels were unchanged in all three phases of trial. According to our knowledge, this is the first study that examined the effect of soy consumption in the form of soy nut and soy protein on serum leptin levels in postmenopausal women with metabolic syndrome. Previous studies on the effect of soy on hormonal changes like leptin have not been conducted on patients with metabolic syndrome. Patients in the current study had all five components of metabolic syndrome, and the leptin level was elevated in these patients. However, the soy products with the natural levels of isoflavone used in the present study could not change the serum leptin level in these patients. It was hypothesized that the amount and the dose of the isoflavone intake was not effective for this aim. Furthermore, our control diet was a DASH diet, which was full of the isoflavones and therefore, this might be the reason for not seeing any effect from soy products’ consumption compared to the control group.

In a rat model, Chen et al^12^ indicated that soy isoflavone might decrease body weight of rats and leptin mRNA, increase serum leptin levels, and ameliorate leptin and insulin sensitivities. Another study on rats also reported an antiobesity effect from the soy peptide consumption by activating the leptin-like signaling and AMP-activated protein kinase.^13^ This was also the case in human studies. A meal replacement diet among humans in which soy products were replaced in two daily meals, significantly reduced serum leptin levels as compared to the control diet.^14^

Based on some experimental investigations, vegetable protein such as soy protein can stimulate satiety and then prevent weight gain^21^,^22^ but clinical trials have not confirmed these results yet.^23^,^24^ Isoflavones, fatty acids, sapon-ins and phospholipids content of soy might have beneficial effects on weight. Soy protein might also affect lipid absorption, insulin resistance, serum leptin level, and other hormonal, cellular, or molecular changes associated with adiposity.^25^ However, further studyes are needed to clarify the effect of soy products consumption on serum leptin levels and weight changes.

Conflicting results in different studies might be due to subject selection, doses of isoflavones intake and even the duration of study. The amount of soy isoflavones participants consumed in the current study was 84 mg/d during the soy protein period and 102 mg/d during the soy nut period. These amounts were lower than the doses used in previous effective trials,^26^,^27^ but higher than the isoflavones content of diets commonly consumed in some Asian countries where soy is a staple food (20-80 mg/day).^27^,^28^

This study was a short-term study on the patients with metabolic syndrome. Although a recent long-term study showed the beneficiary effects of soy on the metabolic parameters of the diabetic patients,^29^ and further confirmed the results of previously conducted short-term studies,^30^ it is suggested to conduct a longitudinal study in this field on patients with metabolic syndrome.

Obesity is a multifactor disease and different dietary pattern and food intake might be responsible for it.^31^–^33^ However, paying attention to the individual food item in the diet also might be important. Therefore, besides considering the role of dietary pattern and food group intake, considering the different food intake with possible role in obesity is also recommended. Soy products are one of the important foods in this regard.

## Conclusions

Although the results of previous studies indicated beneficial effects of soy consumption on the cardio-metabolic abnormalities, the results of the present study showed that neither soy protein nor soy nut could effect weight or serum leptin levels in postmenopausal women with metabolic syndrome.
